# Listening and Hearing: A Voice Hearer's Invitation into Relationship

**DOI:** 10.3389/fpsyg.2017.00387

**Published:** 2017-03-14

**Authors:** Berta Britz

**Affiliations:** Berta Britz ConsultingHavertown, PA, USA

**Keywords:** lived experience, hearing voices, adverse childhood experiences

## Abstract

Although historically overlooked, empirical links between trauma and psychosis have received growing attention over the past decade. Increasingly, clinical researchers have also zeroed in on the role that distressing or traumatic life events play in the psychosocial formation and maintenance of psychosis. This paper re-locates anomalous experiences in their human contexts, and asks that clinicians and researchers engage with these contexts. The author shares a first person account of her experience changing her relationships with dominance in order to reclaim and accept her human being-ness, a reorientation supported by her involvement in the world hearing voices network movement and community. She calls for mental health systems, providers, and researchers to collaborate with the persons at the center of their work—to dare to listen, hear, and connect for mutual learning, healing, and wholeness. The article concludes with recommendations and a rallying call for services to be made more inclusive and to re-center in meaningful collaboration with people with lived experience. More comprehensive, meaningful, and accountable practices can be co-created when people are met equally as human subjects, both responsible and accountable for change.

## Conceptual frame

The prevalence of trauma—adverse childhood and adult experiences—in the lives of the people served in the mental health system has been well-established (Álvarez et al., 2011; Larsson et al., 2013). Although, it has been widely acknowledged that providing high quality mental health services requires a trauma informed approach, actual practice is inconsistent, and often absent in clinical work with persons from marginalized and/or under-represented groups (culturally, racially, economically, sexually), including those diagnosed with psychosis (Muskett, 2014; Read et al., 2016). In the area of psychosis specifically, the evidence on more conventional trauma-driven therapeutic approaches such as cognitive behavioral therapy and EMDR for persons with psychosis remains startlingly sparse (Bendall et al., 2010). In contrast, trauma-driven approaches often dominate the landscape of peer-led, peer-informed, and “alternative” approaches to psychosis, including the body of supports and techniques developed within the international hearing voices movement (Longden et al., 2012; Corstens et al., 2014). A core tenet of these latter approaches is that the genesis, messages or content, and phenomenology of voices and unusual beliefs are often, if not always, strongly interconnected with individuals' personal histories and distressing or traumatic life events (Corstens et al., 2014). The hearing voices movement, and allied approaches, strongly encourage the exploration of the meaning of voices and beliefs, and the re-centering of the relationship between the individual and his or her voices/experiences. Phenomenologically-informed work more broadly attests to the high prevalence of voices and other experiences that are subjectively perceived as rich, meaning-laden, interconnected with life events, and with whom the individual engages as he or she would a person, character, or entity (Woods et al., 2015).

This seeming disconnect between peer-driven approaches and conventional (particularly pharmacotherapy-driven) clinical approaches may stem from the illness-focus and power imbalance in the relationships between clinicians, researchers, and individuals using services. Both clinical service systems and the research that supports them have traditionally strongly marginalized, or outright excluded, the voices and perspectives of persons with lived experience of psychosis (Faulkner and Thomas, 2002; Callard et al., 2012).

Research demonstrates that clinicians often think diagnostically (what's wrong with you?) when approaching clients; however when thinking about their own pain/distress, they think about its context (what's happened to them; Carter et al., 2016; Magliano et al., 2016). Clinical training teaches us to ask and look for presenting problems, focusing on the deficits in the individual or, more rarely, in the family/social system. In typical practice settings in the USA, the structure of the diagnosis, treatment, and documentation upon which payment depends relies on a clinical lens shaped by presuppositions that are illness-focused rather than person-centered, thereby reducing the likelihood of clinicians engaging relationally with the persons they seek to help. The following essay seeks to shift the conventional expert/client perspective to enable a relational lens based in curiosity and listening that values the experiences of both humans at the clinical table.

## Experiential lens

*Today I am a woman firmly planted in this world—I belong! Mine has been an odyssey from fear, shame, hopelessness, and “psychosis” to home and liberation. Everything I understand involves relationship to other. The other inside me, the other outside me, the meaning I make and co-create. I am responsible for myself, and I am inextricably connected to my ancestors, my family of origin, the people, and animals I know and have known, nature, community, and Spirit. My experience of disconnect, chaos, and powerlessness, labeled “psychosis,” makes sense in the context of those relationships. My journey toward understanding began steeped in fear and terror, fueled by my drive to survive and my thirst for meaning. Like Odysseus I faced opposition and oppression. At times my vessel sank in quicksand, battered by riptides and rocks, I continually emerged to reorient and breathe. In relationship I moved toward healing and wholeness*.

*I will share my current understanding of how I am becoming whole and its possible relevance to others. I've learned from being part of several communities—the community of voice hearers, Quakers, people working for individual mental health and mental health systems recovery, people working for social and restorative justice and to preserve our planet. I start with my own individual experience of powerlessness and describe the process of moving from aloneness and alienation to connection and love. One of my earliest limiting beliefs was that I was alone and needed to stay that way for my own survival and for the survival of other people. While that might have made a type of sense at adverse times in my early life, it was an anachronistic belief that limited my contacts and possibilities for human connections, learning, and growth over time. As a young child I was afraid of my vulnerability and denied it in order to appear stronger and safer than I felt*.

*My recovery, my rebirthing, has been a process culminating in my current condition of “emancipation.” My emancipation encompasses freedom from old identities forged from fear—my own personal fears and the impact of fear-based interactions with others, especially with others in the mental health/illness systems. I now affirm my birthright as a human being, midwifed by my spiritual, emotional, psychological, and communal relationships with others. I am living into a current freedom to contribute to creating a more just, sustainable community. My current state is a new embodiment of responsibility for myself in relation*.

*The people I depended on as an infant and young child did and said things that helped as well as harmed me. My early life felt confusing and terrifying, and it was my connection with animals that sustained me. I lacked basic trust and hid my vulnerability*.

*My identity was forged in powerlessness. My parents tried and failed to save relatives from Nazi concentration camps, and my birth coincided with my mother's grief and rage at the death of her mother. My older brother who had been a caregiver for our maternal grandmother, was reassigned to take care of me, and his care for me included sex. I grew up worshiping my mother and brother, absorbing a mixture of learned helplessness mixed with exceptional power. Surviving was a burden for us all*.

*When I was an adolescent my disconnection was palpable, and the psychiatric system labeled me psychotic, my voices “auditory hallucinations.” My verbalization didn't work; I spoke little and when I did speak or write, it was mixed up. Words meant different things to me. I didn't speak directly—my thoughts and beliefs were dangerous, embodied in terrorizing voices. I connected in code with the people I encountered, and my voices used their own codes to both command and obfuscate. For over 40 years my voices echoed and amplified the harsh and intrusive messages I received in childhood. I believed that I wasn't human. It was declared by my voices and also confirmed and reflected in the objectifying way the psychiatric system conceived and treated me*.

*I swallowed the beliefs of my voices and the assumptions of the psychiatric system. Whether my badness stemmed from what my voices considered my substance or from psychiatry's ascription of it to my genes and biochemistry, most things were my fault. I felt battered and assaulted by voices and welcomed damage control from medical experts. Terror imprisoned me, and I found belonging and “safety” in hospitals and in psychiatry's dominant message for fixing or at least managing me*.

*My voices intoned, “You belong in flames. Set yourself on fire.” As a toddler I was lifted high in the air and threateningly shown flames in the kitchen incinerator. Throughout my childhood I learned from the stories about Nazi ovens. As a young girl my mother told me that I caused her suffering and illness. Years later my voices said, “All that you touch is tainted.” “Stab the eyes, slash the arms.” Voices preyed on my fear—claimed to be all powerful and all knowing. They embodied my rage and powerlessness. I was afraid of my voices, afraid of myself. Well-intentioned psychiatrists tried to shut down those voices without considering or acknowledging that the messages might mean something to me. I was told that my experience was not real, and the psychiatric system would help me by annihilating my “symptoms,” the messengers. Dominant professionals battled dominant voices, and my relationship with all was subordinate and powerless*.

*Much treatment unwittingly reenacted hurtful experiences from my childhood. I voluntarily accepted huge doses of neuroleptic medications, but even with that treatment compliance, I acted on the commands of voices to do violence to myself and others. My body was held down, stripped, injected, restrained, and kept in seclusion rooms. I wasn't allowed to use the bathroom and, when in cold wet packs, I had to lay in my urine. At intervals my breathing was monitored, and no one spoke to me for hours. I was denied the experience of being held as a human being and acknowledgment of my right to be. In the 1960's and 1970's I was grateful that the psychiatric system fought to control my dangerousness to protect me and others. I did not want to hurt anyone. I considered psychiatry's aggressive tactics ethically warranted, just as I had accepted the necessity of our country's going to war to fight Nazis*.

In the decades that followed, the psychiatric system's solutions for my “behavior” have remained essentially unchanged. They stopped using cold wet packs with me, sometimes provided bedpans, and continued advertising new generations of miracle medications and promising treatments. I have voluntarily used most of their tools. Conventional psychiatry continues to judge my experience as not real. They see my “symptoms” as random, arbitrary effects of neurotransmitters, and genetics, and their well-intentioned goal is my adherence to medication treatment, avoidance of hospitalization, and “maintenance.” I no longer accept such invalidation. I have chosen a different path, and my psychiatrist has told me that he expects me to decompensate. If I don't, then the only explanation that his belief system can accommodate is that my diagnosis must have been wrong—wrong for half a century, yet it is I who lack capacity for insight!

*He and others in the psychiatric system view me as a defective object to be fixed, and our society has accorded them the role of fixer. Profitmaking and fear-based policies, not scientific rigor or compassion propel our current approach to human suffering. I no longer wear the mantle of “other” as a shackle. I have re-oriented to locate myself, centered to meet, and connect with other humans, nature, and Spirit in respectful relationship. I honor the process of listening, hearing, and expressing together. I don't have solid answers. I have trust that through asking questions with open minds and open hearts, the asking will carry us forward together. Rather than battling pain, fear, and conflict as “other,” we can move through the dissonance to seek and co-create multi-dimensional possibilities that include and value all beings, all voices*.

*It was only after struggling to combat fierce voices for over forty years that I discovered the World Hearing Voices Network Movement. By assertively changing my relationship with my voices, I moved from feeling powerless and disconnected to discovering and affirming their meaning and learning to accept acceptance. Joining the HVNM was not my first liberating experience, but it was qualitatively different from my other healing experience where I had learned that I could live “as-if” I was human. In the 1970's a unique psychologist nurtured me, taught me to show facial expression, to reconnect with common language, and rekindled hope that I could continue growing, learning, even loving. Even in this relationship I learned that to find a place in society, I would need to continue keeping secrets and fight against my anomalous experiences and beliefs. I found meaning working with children who were suffering but learned that I had to conceal my own pain and psychiatric experiences in order to progress through school, training, and professional education. I tried to blend into a system that would view me as “less than human” if I allowed myself to be seen and known. I was incapable of sustaining the contradictory demands of being authentic in relationship with children while denying my deepest self. The harder I tried to suppress my extreme experiences, the more intrusive and overwhelming they became. I accepted disability, hopelessness, and helplessness. Then I stumbled into the Recovery Movement which taught me that I didn't have to conquer my “symptoms” before I could engage in meaningful work. But my subordinate relationship to my voices and beliefs impeded that capacity. When I returned to work in mental health recovery, I still accepted the dominance of my voices and their messages. When I was asked to support another voice hearer, I knew that I must find another approach since mine had not worked. That propelled me to Intervoice and Working to Recovery via the internet, a new medium for me. I connected with experts by training and experts by experience in other countries and connected my local community with them as well. It was in learning and growing together in trainings and in developing Taking Back Our Power Hearing Voices Groups that I grew into my birthright as a human. Finally, I developed my right and responsibility for my own being, belonging, believing, and becoming. I shared my developing agency with others, and we grew together in community. During this time I also grew into my spiritual home with my Quaker Meeting where I realized that moving from “power over” to “power with” was essential, and it led me to recognize “power within.” That mutual liberation has continued nurturing me and our local Taking Back Our Power groups and our Hearing Voices Learning Community and Hearing Voices Network*.

*I know that love helps, and hatred hurts. The major barrier that I've encountered to love has been fear, both individual and societal fear. It has helped me to examine power and powerlessness—actual and perceived. My liberation has involved changing how I relate to “otherness”—the outside world, other cultures, people, adversities, and to my own experience of “otherness”—my voices, visions, anomalous beliefs, fears, and rage—the “other” within and the “other” without. I couldn't make those shifts in my relationships and in my perception of power and powerlessness by myself alone. I needed to do it in relationship, the same way I came into the world at birth, and the same way I developed as a child and adult, even when I considered myself totally alone. Feeling alone relates to presence as well as absence. In recent years, I have learned to meet and engage with “other,” to befriend my whole self and open opportunities for building a more compassionate, inclusive community. I believe that a similar process of “being-with” to understand “other” is a process by which we can effect both individual and social change—a way to change our relationships with dominant voices/powers within our cultures, to embrace diversity and take steps toward sustaining our relationships with each other and the planet*.

*Judi Chamberlin's admonition, “Nothing about us without us” taught me and countless others the importance of finding and using our voice. I have been trying to make sense of my life for as long as I can remember. I have either received, used, provided, or connected with mental health services for over 50 years. Currently I connect with individuals, groups, and systems to promote listening to all experiences and supporting people in lifting their voices to promote mutual understanding and choices for healing and wholeness. When Judi Chamberlin was dying, she wrote that her experience in hospice was the closest to the person-centered “being-with” that she had advocated for in mental health services (Goldberg, 2009). My plea today is for mental health professionals to join in such human accompaniment. We need not wait for hospice: we can create spaces that honor our individual and collective journeys—no matter how painful or frightening. We do this together by practicing presence with courage, curiosity and love*.

## Unifying vision

Historically, gaps between the experiences of service users and clinicians have all too often led to unnecessary suffering and exclusion, as we see amply illustrated in the history of forced long-term institutionalization, hydrotherapy, involuntary sterilization, and prefrontal lobotomies (Mechanic and Rochefort, 1990; Braslow, 1999). While it is easy to dismiss past practices as naïve and unparalleled in contemporary practice, Braslow (1999) underscores how many of such practices were defended by renowned researchers, including two Nobel laureates in Medicine and the “state of the science” of the day. If randomized controlled trials are one way of working to ensure accountability within systems of care, service user involvement, and leadership is at least as important. Researchers and clinicians, that is, must not only engage with service users' experiences, but collaboratively investigate and interrogate extant understandings of “psychosis” and its causes and origins, practices that support healing, and to deconstruct the power dynamics and hierarchies that continue to dominate the production of knowledge and qualification or disqualification of different forms of experience and expertise (Kalathil and Jones, 2016).

## Author contributions

The author confirms being the sole contributor of this work and approved it for publication.

### Conflict of interest statement

The author declares that the research was conducted in the absence of any commercial or financial relationships that could be construed as a potential conflict of interest.
